# Beyond the Rhythm: In Silico Identification of Key Genes and Therapeutic Targets in Atrial Fibrillation

**DOI:** 10.3390/biomedicines11102632

**Published:** 2023-09-25

**Authors:** Natalia Atzemian, Nikolas Dovrolis, Georgia Ragia, Konstantina Portokallidou, George Kolios, Vangelis G. Manolopoulos

**Affiliations:** 1 Laboratory of Pharmacology, Medical School, Democritus University of Thrace, 68100 Alexandroupolis, Greece; ntatzemi@med.duth.gr (N.A.); gragia@med.duth.gr (G.R.); konporto@affil.duth.gr (K.P.); gkolios@med.duth.gr (G.K.); 2 Individualised Medicine & Pharmacological Research Solutions Center (IMPReS), 68100 Alexandroupolis, Greece; 3 Clinical Pharmacology Unit, Academic General Hospital of Alexandroupolis, 68100 Alexandroupolis, Greece

**Keywords:** atrial fibrillation, bioinformatics, fibrosis, atrial remodeling, transcriptomics, proteomics

## Abstract

Atrial fibrillation (AF) is a prevalent cardiac arrhythmia worldwide and is characterized by a high risk of thromboembolism, ischemic stroke, and fatality. The precise molecular mechanisms of AF pathogenesis remain unclear. The purpose of this study was to use bioinformatics tools to identify novel key genes in AF, provide deeper insights into the molecular pathogenesis of AF, and uncover potential therapeutic targets. Four publicly available raw RNA-Seq datasets obtained through the ENA Browser, as well as proteomic analysis results, both derived from atrial tissues, were used in this analysis. Differential gene expression analysis was performed and cross-validated with proteomics results to identify common genes/proteins between them. A functional enrichment pathway analysis was performed. Cross-validation analysis revealed five differentially expressed genes, namely *FGL2*, *IGFBP5*, *NNMT*, *PLA2G2A*, and *TNC*, in patients with AF compared with those with sinus rhythm (SR). These genes play crucial roles in various cardiovascular functions and may be part of the molecular signature of AF. Furthermore, functional enrichment analysis revealed several pathways related to the extracellular matrix, inflammation, and structural remodeling. This study highlighted five key genes that constitute promising candidates for further experimental exploration as biomarkers as well as therapeutic targets for AF.

## 1. Introduction

Among cardiac arrhythmias, atrial fibrillation (AF) is the most common type worldwide, and its pathophysiology is not yet fully understood. It consists of a significant burden on healthcare systems, and it is considered a “growing epidemic” [1,2] since, nowadays, more than 37 million people worldwide suffer from it, and this number is projected to grow in the next few decades [3,4]. The incidence of AF increases with age, and AF is also the most frequent cause of ischemic stroke incidents in the elderly [5]. The risk factors for AF include comorbidities such as hypertension, congestive heart failure, coronary artery disease, valvular heart disease, and diabetes mellitus. These conditions may induce AF by promoting atrial pressure and dilation [6,7]. AF contributes to cardiac morbidity and mortality since its major complications are thrombosis and thromboembolism, which are often life-threatening [8,9]. 

Despite its prevalence, the therapeutic arsenal of clinicians to treat AF is limited due to inadequate knowledge of its pathophysiological mechanisms, so to date, there is still no effective treatment to improve the prognosis of AF patients [10,11,12]. To date, AF management is based on symptom alleviation with medications, such as direct oral anticoagulants (DOACs), which are used as the cornerstone treatment for stroke prevention in AF. They offer a prominent therapeutic strategy but also exhibit a risk of serious adverse bleeding complications [13]. Other treatments, such as amiodarone and β-blockers, are also used in AF management for rate and rhythm control, but with limited efficacy and several adverse reactions and contradictions [14,15]. Hence, understanding the molecular causes of AF would be beneficial for its effective prevention and treatment, not just alleviating symptoms.

AF is a progressive disease because it usually manifests as paroxysmal events that progress into long-term morbidity, leading to chronic, persistent, and subsequently permanent AF [16]. This progression has been linked to changes in the structure and function of the atria due to extensive structural, contractile, and electrical remodeling, rendering the arrhythmia constant and more long-lasting [16,17,18]. However, the mechanisms and factors that govern AF progression are far from clear. 

Bioinformatics in cardiovascular research have helped uncover the molecular mechanisms of heart disease and reveal new pharmacological targets [19,20,21]. The role of atrial remodeling in AF has already raised scientific awareness. Dysregulation of molecular pathways has been proposed to cause the progression of atrial remodeling and, subsequently, AF [22]. Prior studies have highlighted certain genes involved in the development of atrial remodeling, such as ion channel genes [9,23], signal transduction molecules [24], as well as genes of the atrial extracellular matrix compartment [25]. Thus, atrial remodeling stands as a potential therapeutic target, but its exact mechanisms have yet to be explored [26].

Despite the progress in identifying the components of the pathophysiological mechanisms of AF, there is further new knowledge to be gained. Herein, based on publicly available transcriptomic and proteomic data, we explored samples from the left atrial appendages (LAA) and right atrial appendages (RAA) cardiac tissue of patients with AF and controls (SR, sinus rhythm). With this in silico approach, we aimed to detect the key genes in AF, elucidate its molecular pathophysiology, and contribute to precision medicine by aiding in AF diagnosis, subtyping, and enhancing our understanding of AF mechanisms.

## 2. Materials and Methods

### 2.1. Data Source

#### 2.1.1. RNA-SEQ Datasets Retrieval

To retrieve publicly available RNA-Seq datasets, we searched for the keyword “atrial fibrillation” in the European Nucleotide Archive (ENA Browser; https://www.ebi.ac.uk/ena/browser/home (accessed on 10 January 2023)) [27]. The option “Study” was used as a filter, while only studies that included human samples were selected. Similarly, for statistical significance, studies that included at least 3 patients with persistent or permanent AF and 3 controls were preferred. The post-operational AF datasets were excluded from the analysis. From the dataset PRJNA531935 only samples derived from persistent AF patients were included in the study. From PRJEB42485 only RAA samples from sustained AF and non-diseased patients were used. Furthermore, datasets containing only samples from the LAA and/or RAA were chosen, which were obtained from patients undergoing open-heart surgeries. 

After screening, the datasets PRJNA526687, PRJNA531935, PRJNA667522, and PRJEB42485 were selected. A total of 24 patients (13 with persistent AF, 5 with permanent AF, and 6 with sustained AF) and 25 controls (SR) were included in our analyses. All raw RNA-Seq data were generated using Illumina sequencing platforms. The demographics and characteristics of the RNA-seq datasets are presented in Table 1.

#### 2.1.2. Proteomics Data Retrieval

Differential protein expression analysis results were retrieved from the study by Liu et al., as the raw data were not provided [32]. The study included 18 LAA samples from patients with mitral stenosis undergoing cardiac surgery, 9 with persistent AF, and 9 with SR. The patient characteristics are presented in Table 2. In each clinical condition, three samples that had been stained were mixed in a pooled sample (pooled sample). Proteomic analysis was performed using high-performance liquid chromatography–tandem mass spectrometry (HPLC–MS/MS), and the results were analyzed using MaxQuant software v2.4.4.0.

### 2.2. Data Processing

#### 2.2.1. Identification of Differential Expression Genes

Differential gene expression analysis was performed via RaNA-Seq (v.1.0, Salamanca, Spain) [33], an open bioinformatics cloud tool for RNA-Seq data, in order to detect differentially expressed genes (DEGs). The ENA Study Accession numbers were used as inputs to RaNA-Seq, which then processed the raw sequences for quality control and quantification. Differential expression analysis of the RNA-Seq datasets was performed using the DESeq2 algorithm on the RaNA-Seq platform. Thresholds of absolute Fold Change (|FC|) ≥ 1.5 and *p*-value < 0.05 were used. All samples were used, both RAA and LAA.

#### 2.2.2. Selection of Differential Expression Proteins

The analysis of differential protein expression was performed by Liu and his group in order to identify the differentially expressed proteins (DEPs). They reported that Pearson’s correlation analysis showed good reproducibility between samples, while the mass accuracy of the AF data met the requirement, and the peptide length distribution agreed with the properties of the tryptic peptides. The Benjamini–Hochberg method was used to calculate the False Discovery Rate (FDR). Proteins considered important and selected for our study had thresholds of FDR  <  0.1 and |FC| values ≥ 2.

#### 2.2.3. Defining the AF Molecular Background

The differentially expressed genes and proteins (DEGs and DEPs) were used to generate a Venn diagram from the Molbiotools.com tools [34]. The intersection shared between DEGs and DEPs constitutes the molecular signature of AF. The results of this join were used for subsequent analyses.

#### 2.2.4. Functional Enrichment Analysis

Functional enrichment analysis of the molecular signature of AF was performed using NetworkAnalyst 3.0 (https://www.networkanalyst.ca/ (accessed on 1 March 2023)) [35]. An enriched protein–protein interaction network (PPI) was created and visualized. Using the official acronyms of the genes defined in the molecular signature of AF as gene input, a PPI network was created by selecting the STRING Interactome database to retrieve interacting genes/proteins, while a confidence score cut-off > 400 and the requirement of experimental evidence were also selected [36]. 

#### 2.2.5. Pathway Enrichment Analysis

Pathway enrichment analysis of the five genes was performed using the Kyoto Encyclopedia of Genes and Genomes (KEGG) database and presented using NetworkAnalyst 3.0. Only pathways with a significance value < 0.05 are presented. Using the ExpressAnalyst web tool (https://www.expressanalyst.ca/ (accessed on 1 March 2023)), the same analysis was performed by selecting Gene Ontology (GO) database to visualize the data from Biological Processes, and Cellular Components. 

**Figure 1:**
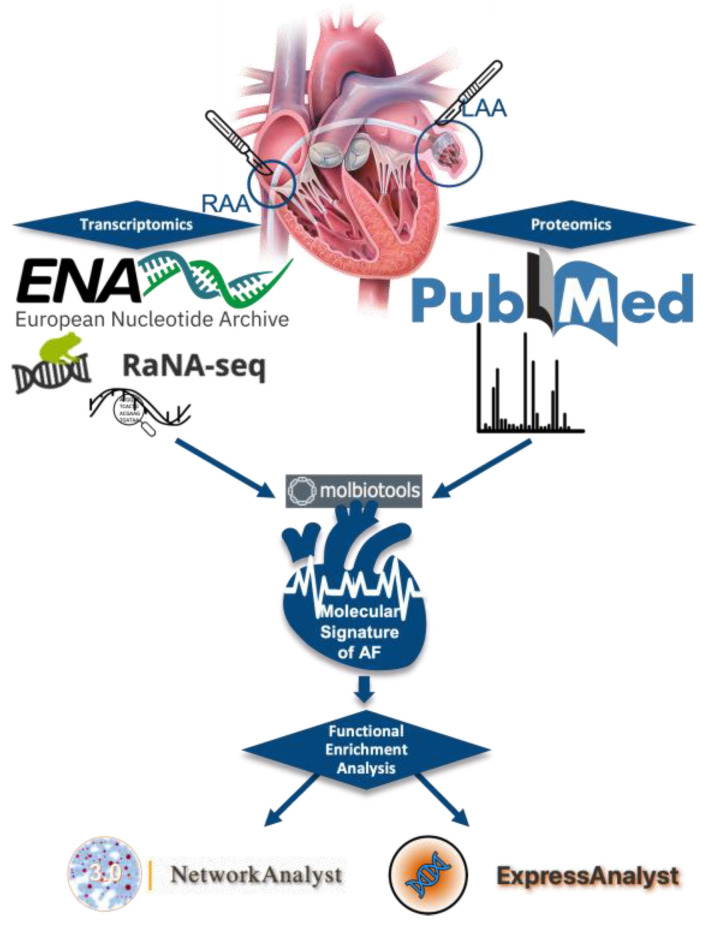
Illustration of methodology steps used in this study. The logos of the platforms used in each step are shown in the figure.

The methodological approach that was followed is summarized in Figure 1.

## 3. Results

### 3.1. Differential Gene Expression Analysis

Using public transcriptional profiles of atrial tissues derived from RNA-Seq experiments, DEGs were identified among the four datasets. A total of 569 genes were found to be differentially expressed with an FC > 1.5 and a *p*-value < 0.05. (Supplementary Materials File S1).

### 3.2. Differential Protein Expression Analysis

The proteomic profile of atrial tissues derived from mass spectrophotometry experiments revealed 59 DEPs. The selection of DEPs was based on FC > 2 and *p*-value < 0.05 (Supplementary Materials File S1).

### 3.3. Defining the Molecular Background of AF

**Figure 2:**
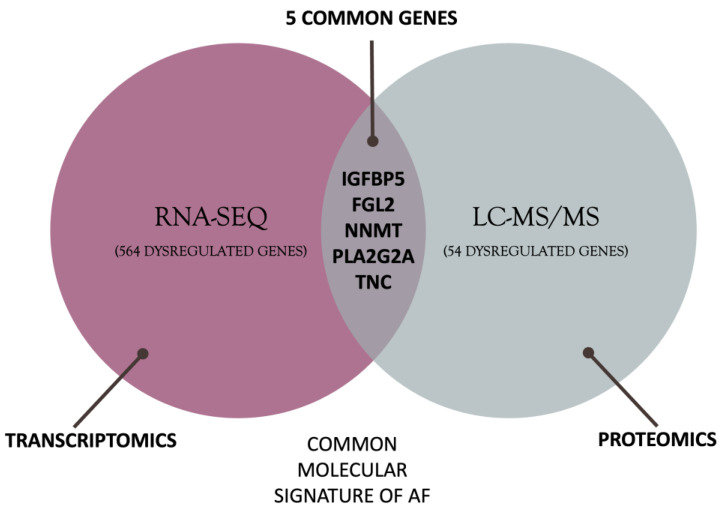
Intersection of RNA-SEQ and proteomics dysregulated genes and the five common genes.

The intersection of DEGs and DEPs revealed five differentially regulated genes in AF patients compared to patients with normal heart rhythm. The five genes are *FGL2* (fibrinogen-like protein 2), *IGFBP5* (insulin-like growth factor-binding protein 5), *NNMT* (nicotinamide N-methyltransferase), *PLA2G2A* (phospholipase A2 group IIA), *TNC* (tenascin C). The intersection defining the molecular signature of AF is shown in Figure 2. 

### 3.4. Functional Enrichment Analysis

**Figure 3:**
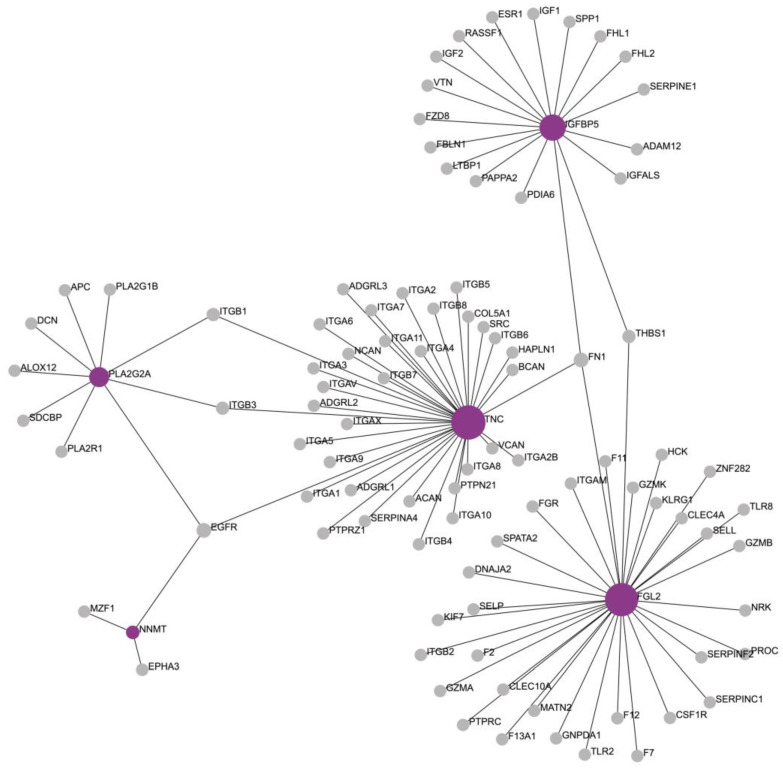
Illustration of the enriched protein network. The molecules of the molecular signature of atrial fibrillation are depicted in purple, while the molecules that enriched the network are in gray.

The molecular signature of AF was used as an input for the functional enrichment analysis and PPI network generation. The generated network consisted of 97 nodes and 99 edges (Figure 3). The network, in addition to the molecular signature, was enriched by NetworkAnalyst 3.0 with another 94 molecules, of which 5 had a degree greater than 1 and betweenness greater than 195: *EGFR*, *FN1*, *THBS1*, *ITGB1*, and *ITGB3* (Supplementary Materials File S1).

### 3.5. Pathway Enrichment Analysis

The formed network was analyzed using the KEGG database, and the pathways involved in this network were identified. A total of 42 pathways emerged, of which 23 had a significance value lower than *p* < 0.001. Specifically, the detected 23 pathways were involved in the regulation of the extracellular matrix, cytoskeleton, and various cardiomyopathies. The pathways are listed in Table 3.

**Figure 4:**
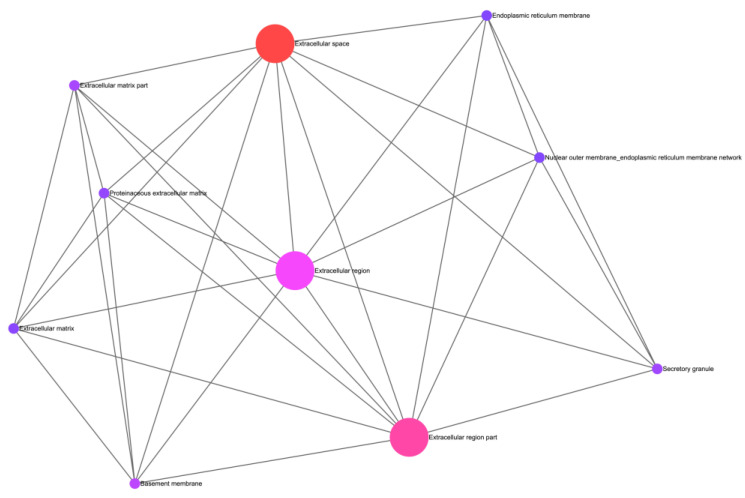
Depiction of the gene ontology cell compartment network in ExpressAnalyst.

**Figure 5:**
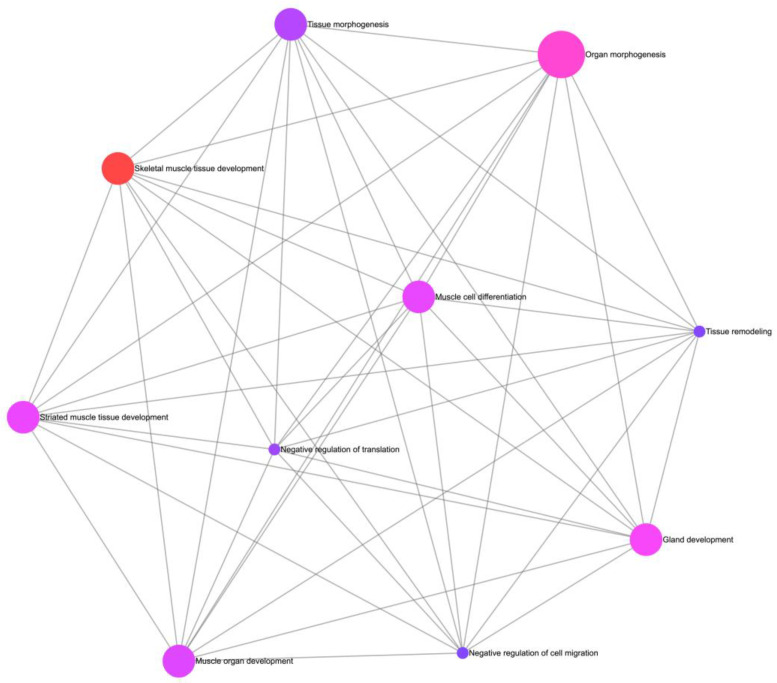
Depiction of the gene ontology biological process network in ExpressAnalyst.

The enrichment of GO terms performed with ExpressAnalyst, from our results, allowed for the generation of two networks that showcase the possible interactions and commonalities between cellular (Figure 4) and functional components (Figure 5). These networks utilize the GO cellular component and GO biological process ontologies, respectively. Figure 4 provides a comprehensive overview of the cellular compartments in which our genes primarily exert their activity within the cell, thus shedding light on their cellular localization. Notably, our analysis highlighted the extracellular region and extracellular space as the most significant cell compartments associated with the five identified genes.

Figure 5 illustrates the biological processes enriched among the identified genes in AF and offers an informative visualization of the processes in which these genes are involved, thereby enhancing insights into the biological mechanisms underlying AF. Specifically, in our analysis, the five identified genes were involved in biological processes such as skeletal muscle tissue development, organ morphogenesis, gland development, striated muscle tissue development, muscle cell differentiation, and muscle organ development.

## 4. Discussion

AF is a complex and multifactorial cardiovascular disease with increasing prevalence. The existing pharmacological approaches to AF are limited because of insufficient understanding of its pathophysiological mechanisms, while early diagnosis and spot-on treatment can improve the prognosis of patients with AF. In recent years, bioinformatics has helped untangle complex pathophysiological mechanisms and discover innovative therapeutic targets in cardiovascular research.

Presently, there is a significant scientific interest in designing novel interventional or pharmacological treatments to prevent or terminate AF. Elucidating the molecular signature of AF through bioinformatics may help reveal innovative therapeutic genes or pathway targets and biomarkers of AF, consequently pursuing precision medicine for AF patients. The nature of AF occurrence and maintenance implicates the complicated regulation of gene expression in atrial tissue. Thus, it is pivotal to understand the mechanisms of AF and gain ground for finding innovative mechanism-based therapeutic approaches. 

In the present multi-omics study, the intersection of RNA-Seq and mass spectrometry analysis led to the identification of five genes that constitute part of the AF molecular signature. A total of 569 DEGs were obtained from RNA-Seq data analysis, and 59 DEPs were identified from mass spectrometry data. The intersection of the two types of expression data highlighted five common genes: *FGL2*, *IGFBP5*, *NNMT*, *PLA2G2A*, and *TNC*. Pathway enrichment and PPI network analyses performed on these genes pinpointed the dysregulated pathways in AF.

The *FGL2* gene encodes a protein involved in both blood clotting and immune system regulation. FGL2 is a protein with both pro- and anti-inflammatory effects [37]. Membrane-bound FGL2 acts as an immunocoagulant molecule that can directly cleave prothrombin to thrombin, which then converts fibrinogen to fibrin, bypassing both intrinsic and extrinsic coagulation pathways [38]. *FGL2* deletion in mice hearts induced early death and dilated cardiomyopathy, while *FGL2* depletion led to ventricular dilatation and remodeling, highlighting the importance of *FGL2* in heart function [39]. This is the first study in human tissues to suggest that the *FGL2* gene may be involved in the pathogenesis of AF. *FGL2* is characterized by complexity and multifunctionality and is involved in cardiovascular diseases and conditions, including the progression of pulmonary hypertension [38], COVID-19-generated thrombosis [40], autoimmune myocarditis, and dilated cardiomyopathy in mice [41], and in an AF porcine model [42].

*IGFBP5* is a member of a family of secreted proteins that bind insulin-like growth factors. It is involved in the regulation of growth and metabolism, as well as vascular smooth muscle cell proliferation [43], and it is expressed during myoblast differentiation [44] and heart development in early embryogenesis [45]. Several pieces of evidence suggest that *IGFBP5* plays a role in heart fibrosis [45,46,47] and porcine AF [48,49]. Moreover, knock-in of *IGFBP5* in mice led to an increase in extracellular matrix protein production that can induce fibrosis [50]. Thus, *IGFBP5* may play a role in the structural remodeling that occurs during the pathogenesis of AF.

This study is the first to find a connection between the *NNMT* gene and AF. *NNMT* encodes an enzyme involved in the metabolism of nicotinamide, a precursor of nicotinamide adenine dinucleotide (NAD+), and an important cofactor in cellular energy metabolism [51]. *NNMT* silencing in human lung fibroblasts downregulates extracellular matrix proteins, and it is proposed as a compelling target for progressive fibrotic disorders [52]. *NNMT* expression is modified in various cardiovascular conditions and participates in oxidative stress, fibrosis, and inflammation, which are two processes implicated in the pathogenesis of AF [47,53,54]. 

*PLA2G2A* encodes phospholipase A2, an enzyme that plays a key role in lipid metabolism, including the breakdown of phospholipids, which are important components of the cell membrane. *PLA2G2A* has been implicated in various biological processes, including inflammation and immunity [55]. Knockout experiments with *PLA2G2A* support its role in proinflammatory disorders [56]. This is the seminal work to correlate its expression with AF. However, there are indications for the involvement of *PLA2G2A* in coronary heart disease and myocardial infarction [57], hypertrophic [58], and dilated cardiomyopathy [59]. 

*TNC* encodes a large extracellular matrix glycoprotein that is mainly expressed during embryonic development and wound healing [60]. *TNC* is a well-studied molecule involved in various cellular processes, such as migration, proliferation, differentiation [61], inflammatory and immune responses, as well as tissue repair and regeneration [60,62]. Specifically, in the heart, *TNC* contributes to extracellular matrix remodeling in response to pathological stimuli, such as ischemia and inflammation, and is involved in the development of cardiac fibrosis [60,61,63]. Dysregulated expression of *TNC* has been associated with various cardiac pathologies, including hypertension, atherosclerosis, myocardial infarction, and heart failure [62,63,64]. Several in vivo studies investigating the functional role of TNC support its proinflammatory and profibrotic action in the heart [65,66,67]. There are no direct associations between *TNC* and AF beyond the present work; however, the evidence that it induces atrial fibrosis and remodeling supports the idea of its contribution to AF pathogenesis.

Together, these genes appear to be involved in the development and progression of AF through various mechanisms, mainly inflammation, fibrosis, and structural remodeling. Concurrently, these genes participate in several pathways, most notably the extracellular matrix-receptor interaction, focal adhesion, and regulation of the actin cytoskeleton. These pathways are interrelated processes that function together to regulate cell adhesion, migration, and survival, while also playing critical roles in tissue growth, wound healing, and cardiac fibrosis [68,69,70,71]. The interplay between extracellular matrix receptors and regulation of the actin cytoskeleton is important for maintaining myocardial structural integrity and contractility [72,73,74]. Focal adhesions are specialized structures that connect the extracellular matrix to the actin cytoskeleton, thereby allowing the transmission of mechanical forces [72,75]. Disruption of these pathways can lead to changes in the cellular structure of cardiomyocytes and potentially contribute to the development of AF [73,75,76].

Hypertrophic cardiomyopathy (HCM), arrhythmogenic right ventricular cardiomyopathy (ARVC), and dilated cardiomyopathy are three different pathways that were found to be involved with AF in our analysis. They constitute clinical conditions that are the result of chronic inflammation and fibrosis [77,78,79] and require further investigation for their role in AF pathogenesis. 

The PI3K-Akt signaling pathway is involved in various cellular processes, including cell survival, proliferation, and growth [80,81]. It has been implicated in the pathophysiology of AF, as activation of this pathway can lead to changes in ion channel expression and electrical remodeling of the atrial [80,82,83,84]. Complement and coagulation cascades have also been shown to be involved in the development of AF [85]. Activation of these pathways can lead to inflammation and fibrosis in the myocardium, which can promote the development and maintenance of AF [85,86].

In summary, these pathways are complex and interconnected, and their dysregulation may contribute to the pathophysiology of AF. Moreover, the five genes identified may serve as noteworthy biological regulators, as their dysregulation has been cross-validated at the transcriptomic and proteomic levels. These genes and pathways deserve further exploration in future studies for their roles in fibrosis, structural remodeling, and cardiac inflammation, as they can serve as biomarkers for predicting the onset and progression of AF. Several multi-omics efforts have been made to address the AF burden by incorporating epigenomics and/or transcriptomics and/or proteomics data [32,87,88,89,90]. Given the burden and complex nature of AF, additional advanced AF translational research, including multi-omics approaches, is pivotal.

By integrating multiple RNA-Seq datasets and proteomic data for cross-validation, this study provides a notable advantage in mitigating the occurrence of false-positive results as well as gaining a more comprehensive understanding of biological processes. Moreover, it is important to acknowledge that differences between transcriptomic and proteomic profiles may arise as a result of post-transcriptional modifications; not all mRNA molecules result in functional proteins, highlighting that alterations in transcriptomes do not necessarily translate into observable differences in the proteome. To the best of our knowledge, this is the first investigation to combine multiple RNA-Seq datasets and proteomic data in a multi-omics approach to identify the molecular mechanisms of AF. Moreover, rigorous statistical analysis using stringent cut-offs ensures that results are less susceptible to random fluctuations.

This study has some limitations that should be considered. As this is an in silico study, mechanistic in vivo and in vitro studies are required to validate the results. Specifically, to qualify as a component of the AF molecular signature, each of the five identified genes should be validated by RT-qPCR in independent clinical samples. Follow-up experiments will either confirm or negate our bioinformatics findings, but in any case, they will provide valuable information on the pathophysiological mechanisms of AF. Such an experimental approach will bridge the gap between in silico predictions and clinical practice applications, thereby improving the reliability and translational potential of our research. There is an inherent bias in using datasets generated by different experimental procedures, which we attempted to reduce by analyzing them separately and looking for commonalities between them. Furthermore, this study incorporated datasets originating from diverse geographic locations and encompassing various racial compositions. It is important to acknowledge that, because of the nature of publicly available datasets, as in this case, it is common for detailed clinical parameters (e.g., medication regimens, individual disease courses, and comorbidities) and demographic characteristics (e.g., age, gender, race, and ethnicity) to be missing from the metadata. This information gap may introduce variability into the expression profiles and potentially limit the depth of our analysis. Although we attempted to include patient demographics and characteristics from published articles associated with these datasets, the unavailability of comprehensive metadata remains a notable limitation that could have significantly enriched our study. However, it is plausible to hypothesize that a considerable proportion of patients shared similar medication regimens and comorbidities, thereby implying the presence of comparable confounding factors across the entire study population.

Furthermore, in bioinformatics studies, the results can only successfully account for the association and not the causation between differentially expressed genes/proteins and disorders. The present bioinformatics study is primarily hypothesis-generating and unfit to determine causal relationships. Bridging the gap between the identification of AF-related genes and their practical application in clinical settings remains a substantial challenge. AF is a multifactorial and intricate condition and the five genes we have identified comprise only a small part of its genetic landscape. In order to gain a deeper comprehension of the interplay between these genes and their collective impact on AF susceptibility, it is pivotal to carry out specifically designed experiments to reveal causations, such as in vivo knock-out/in of the genes identified in laboratory animals. Finally, the molecular pathways in which these genes participate have yet to be fully explored and still remain far from translation into clinical practice.

## 5. Conclusions

This study defined a part of the molecular signature of AF using bioinformatics tools. By incorporating transcriptome and proteome data, we highlighted five genes as crucial regulators of AF pathophysiology: *FGL2*, *IGFBP5*, *NNMT*, *PLA2G2A*, and *TNC*. The proposed genes and pathways should be further validated with in vitro and in vivo experimental models, which may lead to major breakthroughs in AF treatment strategies. Overall, this study provides new insights into the molecular pathogenesis of AF and suggests several new promising genes that may serve as therapeutic targets and biomarkers to contribute to the prevention and treatment of AF, improve patients’ quality of life, and reduce the burden of AF on society.

## Figures and Tables

**Table 1:** RNA-SEQ Dataset demographics and characteristics.

Dataset	Samples	Age (Years)	Sex M/F	Country	Tissue Type	Platform	Reference
PRJNA526687	5 Permanent AF	73.6 ± 5.12	5/0	United Kingdom	Paired LAA and RAA	GPL18573	[28]
	5 SR	62.4 ± 6.87	5/0				
PRJNA531935	3 Persistent AF	57 (51–64)	2/1	China	LAA	GPL20795	[29]
	3 SR	39 (38–42)	2/1				
PRJNA667522	10 Persistent AF	53 ± 8	5/5	China	LAA	GPL16791	[30]
	10 SR	55 ± 8	6/4				
PRJEB42485	6 Sustained AF	67.5 ± 1.7	6/0	Germany	RAA	GPL20301	[31]
	7 SR	55.8 ± 8.3	4/3				

**Table 2:** Proteomic data, demographics, and characteristics.

Samples	Age (Years)	Sex M/F	Country	Tissue Type	Platform	Reference
9 Persistent AF	55.5 ± 9.0	4/5	China	LAA	Agilent 300Extend Q/ExactiveTM Plus (Thermo)	[32]
9 SR	50.5 ± 6.5	5/4				

**Table 3:** Pathways from pathway enrichment analysis using the KEGG database.

KEGG Pathways	Total	Expected	Hits	*p*-Value	FDR
Extracellular matrix (ECM)–receptor interaction	82	0.784	25	1.84 × 10^−32^	5.85 × 10^−30^
Focal adhesion	199	1.9	28	3.2 × 10^−26^	5.08 × 10^−24^
Regulation of actin cytoskeleton	214	2.05	28	2.59 × 10^−25^	2.75 × 10^−23^
Hypertrophic cardiomyopathy (HCM)	85	0.813	21	4.95 × 10^−25^	3.48 × 10^−23^
Arrhythmogenic right ventricular cardiomyopathy (ARVC)	72	0.689	20	5.47 × 10^−25^	3.48 × 10^−23^
Dilated cardiomyopathy	91	0.87	21	2.42 × 10^−24^	1.28 × 10^−22^
PI3K-Akt signaling pathway	354	3.39	30	1.46 × 10^−21^	6.63 × 10^−20^
Complement and coagulation cascades	79	0.756	13	3.00 × 10^−13^	1.19 × 10^−11^
Proteoglycans in cancer	201	1.92	17	3.55 × 10^−12^	1.25 × 10^−10^
Cell adhesion molecules (CAMs)	146	1.4	14	6.7 × 10^−11^	2.13 × 10^−9^
Hematopoietic cell lineage	97	0.928	10	2.22 × 10^−8^	6.42 × 10^−7^
Phagosome	152	1.45	11	1.69 × 10^−7^	4.48 × 10^−6^
Pathways in cancer	530	5.07	17	7.3 × 10^−6^	0.000179
Rap1 signaling pathway	206	1.97	10	2.34 × 10^−5^	0.000531
Small cell lung cancer	93	0.889	7	2.7 × 10^−5^	0.000572
Bladder cancer	41	0.392	4	0.000597	0.0119
Leishmaniasis	74	0.708	5	0.000673	0.0126
Platelet activation	124	1.19	6	0.00114	0.0197
Malaria	49	0.469	4	0.00118	0.0197
Bacterial invasion of epithelial cells	74	0.708	4	0.00536	0.0851
Pertussis	76	0.727	4	0.00589	0.0891
Ras signaling pathway	232	2.22	7	0.0065	0.094
Tuberculosis	179	1.71	6	0.00711	0.0983

## Data Availability

Datasets available on ENA Browser (https://www.ebi.ac.uk/ena/browser/home) Accession Numbers: PRJNA526687, PRJNA531935, PRJNA667522, PRJEB42485.

## Supplementary Materials

The following supporting information can be downloaded at https://www.mdpi.com/article/10.3390/biomedicines11102632/s1.
